# Single cell analysis reveals a subset of cytotoxic-like plasmacytoid dendritic cells in people with HIV-1

**DOI:** 10.1016/j.isci.2023.107628

**Published:** 2023-08-12

**Authors:** Lamin B. Cham, Jesper D. Gunst, Mariane H. Schleimann, Giacomo S. Frattari, Miriam Rosas-Umbert, Line K. Vibholm, Renée M. van der Sluis, Martin R. Jakobsen, Rikke Olesen, Lin Lin, Martin Tolstrup, Ole S. Søgaard

**Affiliations:** 1 Department of Infectious Diseases, Aarhus University Hospital, 8200 Aarhus, Denmark; 2 Department of Clinical Medicine, Aarhus University, 8200 Aarhus, Denmark; 3 Department of Biomedicine, Aarhus University, 8000 Aarhus, Denmark

**Keywords:** Immunology, Components of the immune system, Cell biology, Transcriptomics

## Abstract

Human plasmacytoid dendritic cells (pDCs) play a central role in initiating and activating host immune responses during infection. To understand how the transcriptome of pDCs is impacted by HIV-1 infection and exogenous stimulation, we isolated pDCs from healthy controls, people with HIV-1 (PWH) before and during toll-like receptor 9 (TLR9) agonist treatment and performed single-cell (sc)-RNA sequencing. Our cluster analysis revealed four pDC clusters: pDC1, pDC2, cytotoxic-like pDC and an exhausted pDC cluster. The inducible cytotoxic-like pDC cluster is characterized by high expression of both antiviral and cytotoxic genes. Further analyses confirmed that cytotoxic-like pDCs are distinct from NK and T cells. Cell-cell communication analysis also demonstrated that cytotoxic-like pDCs exhibit similar incoming and outgoing cellular communicating signals as other pDCs. Thus, our study presents a detailed transcriptomic atlas of pDCs and provides new perspectives on the mechanisms of regulation and function of cytotoxic-like pDCs.

## Highlights

• Single-cell RNA-seq of human pDCs revealed four distinct pDC subsets
• pDC1, pDC2, cytotoxic-like pDC and exhausted pDC subset
• The cytotoxic-like pDCs expressed classic pDC genes, antiviral and cytotoxic genes
• Cytotoxic-like pDCs are distinct from NK and T cells

## Graphical abstract

**Figure:**
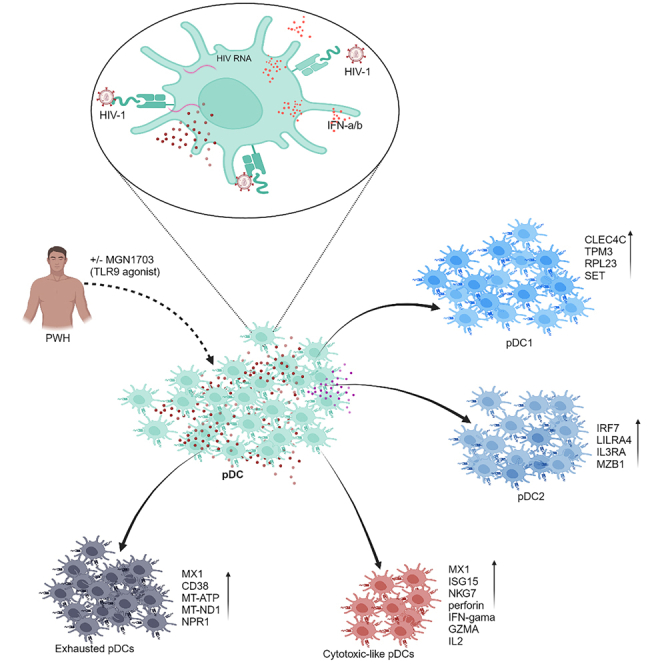

## Introduction

Plasmacytoid dendritic cells (pDCs) are specialized in pathogen sensing and are essential for shaping innate and adaptive immune responses. pDCs rapidly secrete antiviral cytokines and coordinate host immune responses in response to infection.^1^^,^^2^^,^^3^^,^^4^^,^^5^ In pDCs, endosomal Toll-like receptor (TLR) 7 and 9 sense single-stranded RNA and unmethylated DNA, respectively. Upon stimulation, TLR7/9 mediated activation results in upregulation of NF-κB and interferon regulatory factor (IRF) 7 expression, which leads to the production of type I interferons (IFN-I), interferon lambda and other antiviral cytokines.^6^^,^^7^^,^^8^ Following secretion, IFN-α binds to a common heterodimeric IFN-α receptor (IFNAR) which is expressed by most cells. IFNAR engagement activates the tyrosine kinase Janus kinase 1 (JAK1) which phosphorylates the signal transducers and activator of transcription (STAT) 1 and 2 pathway resulting in the transcription of IFN-stimulated genes (ISG).^9^^,^^10^ These ISG-encoded proteins establish an antiviral state in both virus infected cells and neighboring cells thereby limiting virus replication and further spread of infection.^11^^,^^12^^,^^13^ In addition to eliciting an antiviral state, secreted IFN-I enhance innate immune responses by promoting monocyte recruitment and differentiation, cytokine secretion, as well as maturation and activation of myeloid dendritic cells (mDCs).^14^^,^^15^ Furthermore, pDC-secreted IFN-α, IL2 and IL12 are key regulators of NK cell activation, proliferation and cytotoxicity.^16^ Finally, IFN-I enhance the activation and expansion of both antigen-specific CD4^+^ T cells and CD8^+^ cytotoxic T cells,^17^^,^^18^ and promote B cells antibody and memory responses.^19^ In linking innate and adaptive immune responses, pDCs can initiate and promote adaptive immune responses both by cytokine secretion which promote antigen presentation^15^ and by differentiation into potent antigen-presenting cells.^20^

The different human pDCs subsets have historically been defined by their surface protein expression and ability to produce high or low amounts of IFN-α. For instance, Matsui et al. categorized human pDCs by CD2 surface expression into two phenotypical and functional distinct subsets, CD2^lo^ and CD2^hi^ pDCs. CD2^lo^ pDCs produce a higher amount of IFN-α compared to CD2^hi^ pDC. In contrast, CD2^hi^ pDCs were shown to uniquely express lysozyme and secrete high amount of IL12p40.^21^ Another study reported that CD2^hi^ pDCs contained a unique population that expressed CD5 and CD81. These CD2^hi^CD5^+^CD81^+^ pDCs did not secret IFN-α but produced other pro-inflammatory cytokines that stimulated B cell activation and induced T cell proliferation.^22^ In addition to the CD2 classification of human pDCs, several reports have identified a subset of activated pDCs that can express the TNF-apoptosis inducing ligand TRAIL. Stimulation with TLR7 and/or TLR9 agonists has been shown to increase TRAIL expression in pDCs.^23^^,^^24^^,^^25^ Although the mechanisms leading to TRAIL expression in pDCs is not fully understood, TRAIL expression can be induced by IFN-α.^25^ During HIV-1 infection, both infectious and noninfectious virions cause TRAIL upregulation on pDCs. TRAIL^+^ pDCs were only found in HIV-1 viremic individuals and not in nonviremic or healthy individuals.^23^^,^^24^

These observations demonstrate that pDCs can be categorized by many different parameters depending on their activation level. However, because of their scarcity in peripheral blood, our understanding of this intriguing immune cell is incomplete and the present classification of pDC subsets is limited by the surface markers used to characterize them. Hence, in-depth characterization of pDCs heterogeneity and function is of major interest given their crucial role in orchestrating antiviral immunity. To bridge this knowledge-gap, we sorted pDCs from both healthy controls and from people with HIV-1 and performed single-cell RNA sequencing (scRNA-seq) to better understand the biological properties and functions of pDC subpopulations at unprecedent resolution. In the present study, we identified four pDC clusters: two homogeneous clusters (pDC1 and pDC2), a Cytotoxic-like pDC cluster and an Exhausted pDC cluster. The Cytotoxic-like pDC cluster was characterized by combined expression of both antiviral and cytotoxic genes and was distinct from NK cells and T cells.

## Results

### Baseline characteristic of people with HIV and healthy controls

For this study, we used samples from four people with HIV-1 (PWH) who participated in a clinical trial, where a TLR9 agonist (Lefitolimod) was administered as immunotherapy.^26^ During the trial, peripheral blood mononuclear cells (PBMCs) and plasma were collected at baseline (before treatment) and during the 4th week of TLR9 agonist treatment. Among these 4 individuals, the median time since HIV diagnosis was 14.5 years, and the median duration of virological suppression (HIV-1 RNA <50 copies/mL) was 9.5 years. Median CD4^+^ T cells count at baseline was 730 cells/μL. The median HIV-1 DNA level was 565 copies/10^6^ CD4^+^ T cells, and the median cell-associated HIV RNA level was 19 copies/10^6^ CD4^+^ T cells. The healthy controls were individuals without HIV (or hepatitis B/C infection). Clinical characteristics of all donors are summarized in Table 1.

**Table 1:** Baseline characteristic of people with HIV and healthy controls

	Age (years)	Sex	Race	Years since HIV-1 diagnosis	Years from HIV-1 diagnosis to ART initiation	ART regimen	Years with plasma HIV-1 RNA <50 copies/mL^a^	CD4^+^ T cell count (cells/μL)	Total HIV-1 DNA level (copies/10^6^ CD4^+^ T cells)	CA-US HIV-1 RNA level (copies/10^6^ CD4^+^ T cells)
**People living with HIV-1**										

ID103	50	Male	White/	14	2	TDF, FTC, ATV/r	11	980	258	25
			Caucasian							
ID109	70	Male	White/	15	7	ABC, 3TC, EFZ	8	730	429	40
			Caucasian							
ID112	36	Male	White/	7	1	TDF, FTC, RPV	5	730	372	1.1
			Caucasian							
ID113	43	Male	White/	22	0	ABC, EFZ,ATV/r	15	420	1,202	11
			Caucasian							

**Disease-free controls**										

ID61	25	Male	White/							
			Caucasian							
ID63	28	Male	White/							
			Caucasian							
ID65	24	Female	White/							
			Caucasian							
ID66	24	Male	White/							
			Caucasian							

ABC, abacavir; ART, antiretroviral therapy; ATV/r, atanavir/ritonavir; CA-US, cell-associated unspliced; EFZ, efavirenz; FTC, emtricitabine; RPV, rilpivirine; TDF, tenofovir; 3TC, lamivudine.

a During suppressive ART ID103 and ID109 have had a blip of 59 and 62 copies/mL, respectively.

### HIV infection modulates the transcriptional profile of human pDCs

**Figure 1:**
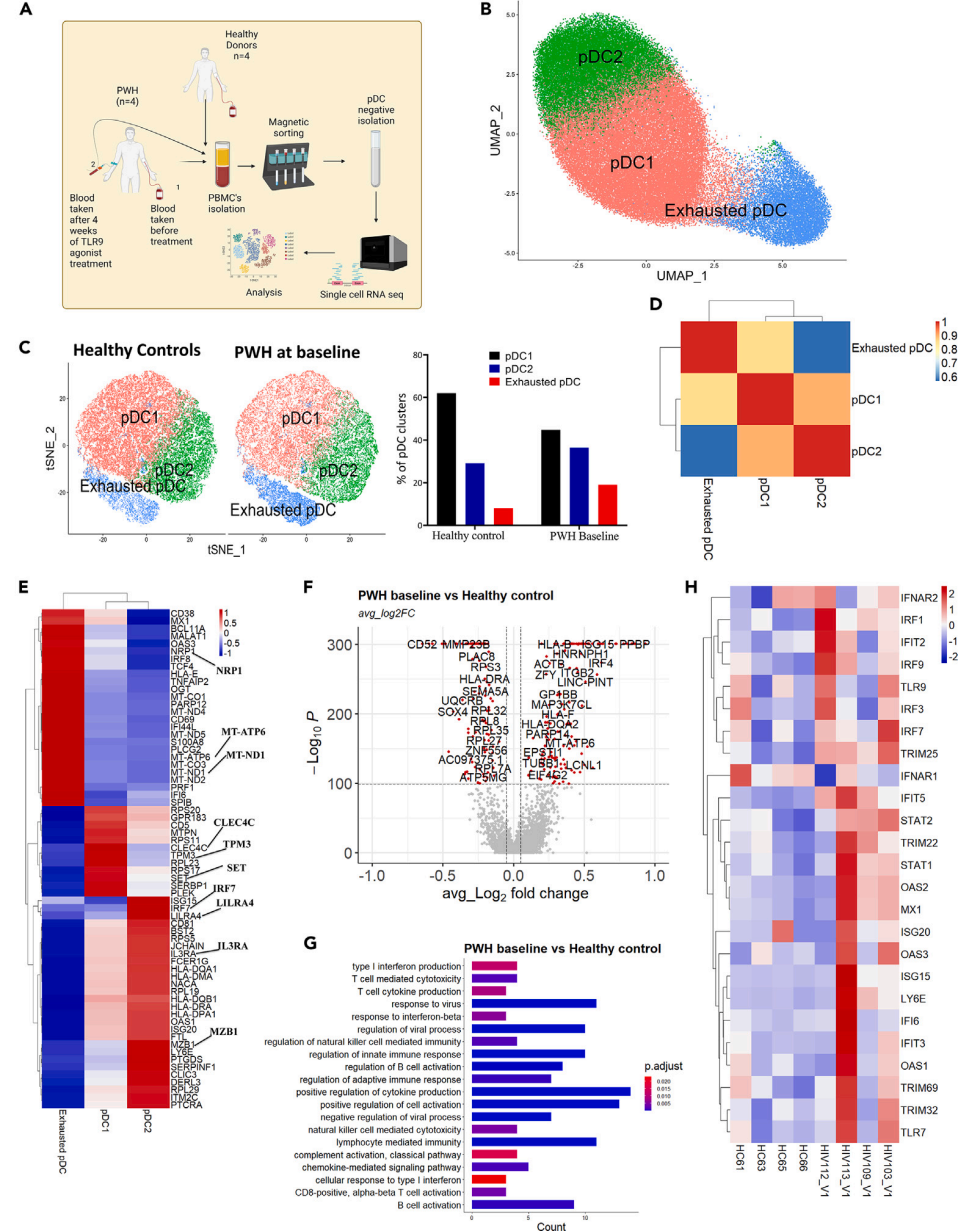
HIV infection mediates transcriptional change in human pDCs (A) Schematic illustration of experimental workflow. pDCs were negatively sorted from four healthy individuals, four PWH on ART at baseline and after 4 weeks of TLR9 agonist treatment. These sorted cells were used to perform scRNA-seq using the 10X Genomic platform. (B) UMAP representation of integrated pDC dataset of (n = 73,549 pDCs) from healthy and PWH at baseline. (C) tSNE plot split cluster and bar plot distribution in healthy and PWH. (D and E) (D) Cluster correlation analysis of pDC clusters and (E) heatmap illustration of relative gene expression level among pDC clusters. (F and G) (F) Volcanos plot of differentially expressed genes (DEGs) and (G) bar plot representation of gene ontology of 100 top DEGs of PWH vs. Healthy individuals. (H) Heatmap representation of relative gene expression of IFN regulatory genes and antiviral genes at individual level. Samples from PWH at baseline are annotated with HIV and V1, HC represent healthy control samples.

To characterize the transcriptome of pDCs at single cell level in healthy individuals and PWH before and during *in vivo* stimulation of pDCs, we first removed dead cells and performed negative isolation of pDCs as shown in (Figure S1A). The isolated cells were used to perform scRNA-seq as shown in the schematic illustration in (Figure 1A).

To determine how HIV infection impacts the transcriptomic profile of pDCs, we integrated the sc-RNA datasets from PWH at baseline and healthy individuals, normalized the integrated gene expression matrix and performed cluster analysis. We identified 13 clusters among which 3 clusters express classical genes known only in pDCs. We annotated these 3 clusters as “pDC” and then annotated the other clusters of other immune cells based on their known gene signatures (Figures S1B and S1C). Next, we selected the pDC cluster from the UMAP plot of the integrated dataset and performed downstream analysis on a total of 73,549 pDCs. After re-clustering, our analysis identified two homogeneous clusters (pDC1 and pDC2) and an exhausted pDC cluster (Figure 1B). Next, we verified their pDC origin and excluded the possible contamination of other immune cells within these three clusters. In all three clusters, pDCs expressed classical gene markers such as *CLEC4C, IL3RA, LILRA4, and MZB1*. Conversely, we found no expression of *CD3G* and *SIRPA* which are known markers for T cells and monocytes/other myeloid cells, respectively (Figure S1D). A study on dendritic cells classification identified a new DC subset called AXL^+^ SIGLEC^+^ (AS) dendritic cells which predominantly express *AXL* and *SIGLEC6* and share properties with pDCs.^27^ However, we observed no expression of *AXL* or *SIGLEC6* within our pDC clusters, thus excluding the possibility of contamination from this AS DC subset (Figure S1E). We then compared the distribution of pDC clusters between healthy controls and PWH. We found that while all three clusters were present in both groups, increased proportions of exhausted pDCs were observed among PWH (Figure 1C). Moreover, cluster correlation analysis and relative gene expressions confirmed a strong correlation between pDC1 and pDC2 clusters (Figure 1D). Gene expression analysis of the pDC clusters revealed that the pDC1 cluster was characterized by relatively high expressions of *CLEC4C, TPM3, RPL23, SET,* while the pDC2 cluster was characterized by relatively high *IRF7, MZB1, HLA-DR, LILRA4* expression. The exhausted pDC cluster had relatively higher expression of genes associated with terminally activation (e.g., *CD69, CD38, MX1, OAS3, IFI6*), apoptosis, mitochondrial stress (e.g., *MT-ND1, MT–ND2, MT-CO3*), and cell exhaustion (e.g., *NRP1*) (Figure 1E).

Next, we sought to interrogate potential HIV associated transcriptomic signatures in pDCs and performed a differential gene expression analysis comparing pDCs from PWH (prior to TLR9 agonist treatment) to those from healthy individuals. We observed pronounced upregulation of *HLA-B, HLA-F,* as well as IFN-α regulatory genes such as *IRF2*, *IRF4*, *IRF7*, and *IRF9* and several antiviral ISGs among PWH compared to healthy individuals (Figure 1F). Gene ontology analysis revealed distinct enrichment of IFN-I production and cellular responses to IFN-I, complement activation and NK cell activation in PWH (Figure 1G). To further decipher the transcriptional signature at the individual level, we analyzed the relative expression of genes associated with regulation of IFN-α production (e.g., *STAT1/2, IRF1, IRF3, IRF7, IRF9*), and antiviral ISGs and we found upregulation of these genes among PWH compared to healthy individuals (Figure 1H). Collectively, we uncovered three pDC clusters and a pronounced upregulation of interferon regulatory genes as well as antiviral ISGs in PWH, even after several years of plasma HIV RNA suppression.

### Cytotoxic-like pDCs expand during TLR9 agonist treatment

**Figure 2:**
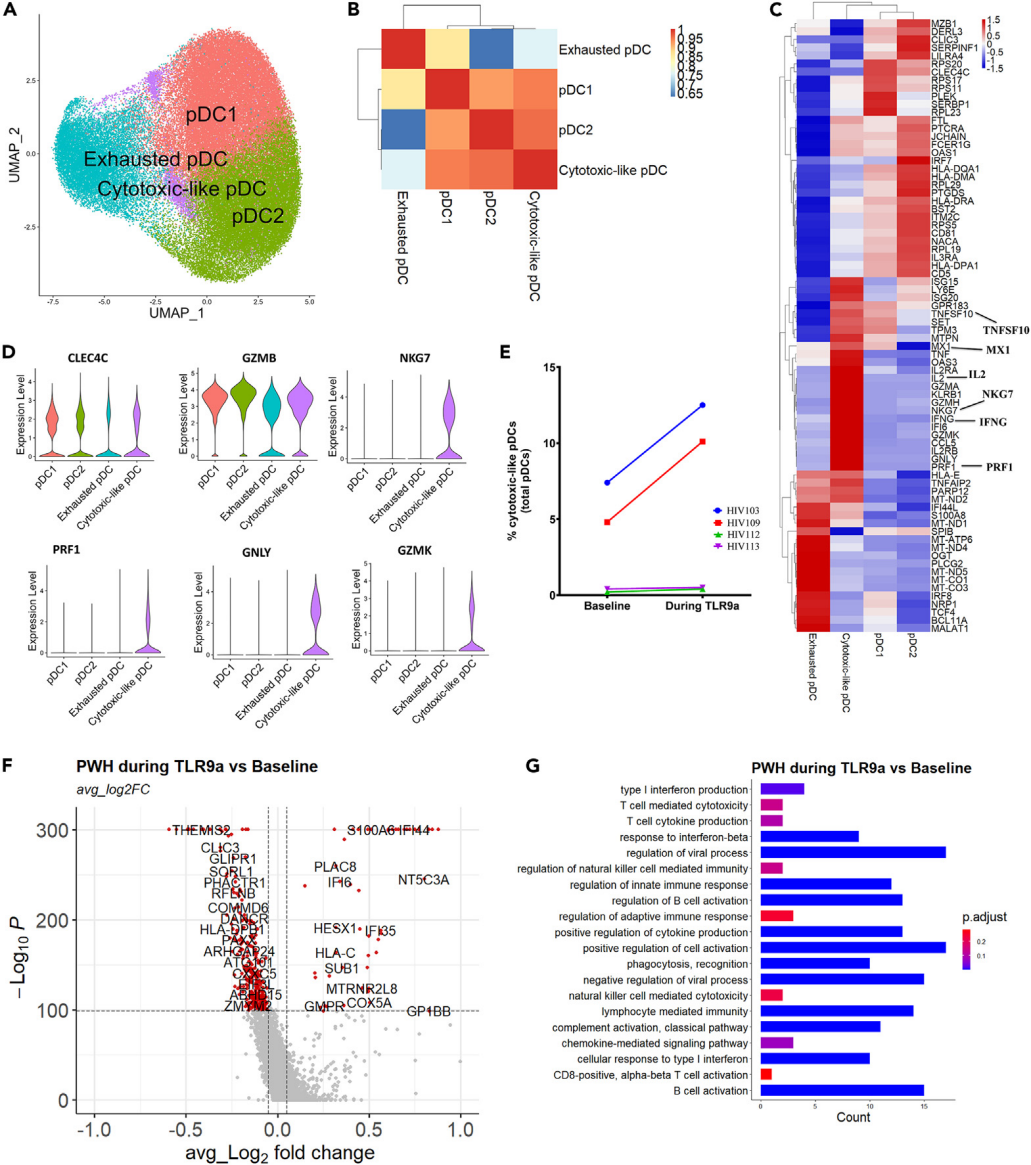
TLR9 agonist treatment induces expansion of a novel cytotoxic-like pDC subset (A) UMAP illustration of integrated pDC dataset (n = 68,032 pDCs) from four PWH on ART at baseline and after 4 weeks of TLR9 agonist treatment. (B and C) (B) Cluster correlation analysis of pDC clusters and (C) heatmap illustration of relative gene expression level among pDC clusters. (D) Violin plot representation of expression of *CLEC4C, GZMB, NKG7, PRF1, GNLY* and *GZMK* among pDC clusters. (E) Percentage of cytotoxic-like pDC cluster at baseline and during TLR9 agonist treatment. (F and G) (F) Volcanos plot of differentially expressed genes (DEGs) and (G) bar plot representation of gene ontology of 100 top upregulated DEGs of PWH before TLR9 agonist treatment Vs. after treatment. Samples from PWH at baseline are annotated with HIV and V1 while after treatment are annotated with HIV and V12.

We have previously shown that TLR9 agonist treatment during HIV-1 infection led to increased activation of pDCs and higher expression of their T cell co-stimulatory markers.^26^^,^^28^ To determine transcriptomic changes during TLR9 agonist treatment at single cell level, we integrated the pDC scRNA-seq data from cells obtained before and during TLR9 agonist treatment among PWH. After quality control, principal component analysis and clustering, we identified 12 clusters and annotated them based on their known gene signatures (Figures S2A and S2B). The pDC cluster was selected for further downstream analysis. Interestingly, our pDC cluster analysis revealed a new cytotoxic-like pDC subset (Figure 2A) which was unlike any of the previously identified pDC clusters (pDC1, pDC2 and exhausted pDC). We employed similar verification and exclusion strategies as above (Figures S1D and S1E) and found no contamination from other immune cells in these pDC clusters (Figure S2C) and no AS dendritic cells (Figure S2D). A cluster correlation analysis found that the cytotoxic-like pDCs had strong correlation to pDC1 and pDC2 clusters (Figure 2B) indicating that they truly are pDCs. However, the cytotoxic-like pDCs had relatively higher expression of antiviral ISGs and cytotoxic genes such as *MX1, ISG15, ISG20, LYE6, OAS3, IFNG, NKG7, GNLY, PRF1, IL2, GZMK, GZMA,* and *TNF,* compared to other pDC clusters (Figure 2C). We further showed that cytotoxic-like pDCs have similar *CLEC4C* and *GZMB* expression, but higher *NKG7, PRF1, GNLY* and *GZMK* expression level compared to other pDC clusters (Figure 2D). We next looked at the frequency of these clusters among our participants and we found that although cytotoxic pDCs were present in all PWH but predominant in 2 out of 4 PWH individuals. HIV103 and HIV109 individuals had higher numbers of cytotoxic-like pDCs at baseline and increased after TLR9 treatment compared to HIV112 and HIV113 individuals (Figure 2E). Of note, we did not identify any individual clinical characteristics such as age, time on ART, HIV-reservoir size, plasma cytokine and chemokine levels that appeared to be associated with the presence of cytotoxic-like pDCs.

Next, we interrogated TLR9 agonist induced pDCs transcriptomic changes by comparing pDCs obtained from before and during TLR9 agonist treatment among PWH. Differentially expressed genes (DEGs) analysis revealed that antiviral ISGs and human leukocytes antigen A, B and C genes were primarily upregulated after TLR9 agonist treatment (Figure 2F). Gene ontology analysis showed that the most enriched biological pathways were involved in regulating CD8^+^ T cell activation, cytokine and chemokine production, NK cell cytotoxicity and B cells activation (Figure 2G). Additionally, we found a global upregulation of both IFN regulatory genes and antiviral ISGs in response to TLR9 agonist treatment (Figure S2E). Overall, we observed TLR9 agonist induced transcriptional changes in pDCs and uncovered a cytotoxic-like pDC subset characterized by a pronounced antiviral and cytotoxic transcriptomic profile.

### Cytotoxic-like pDCs are distinct from NK and T cells

**Figure 3:**
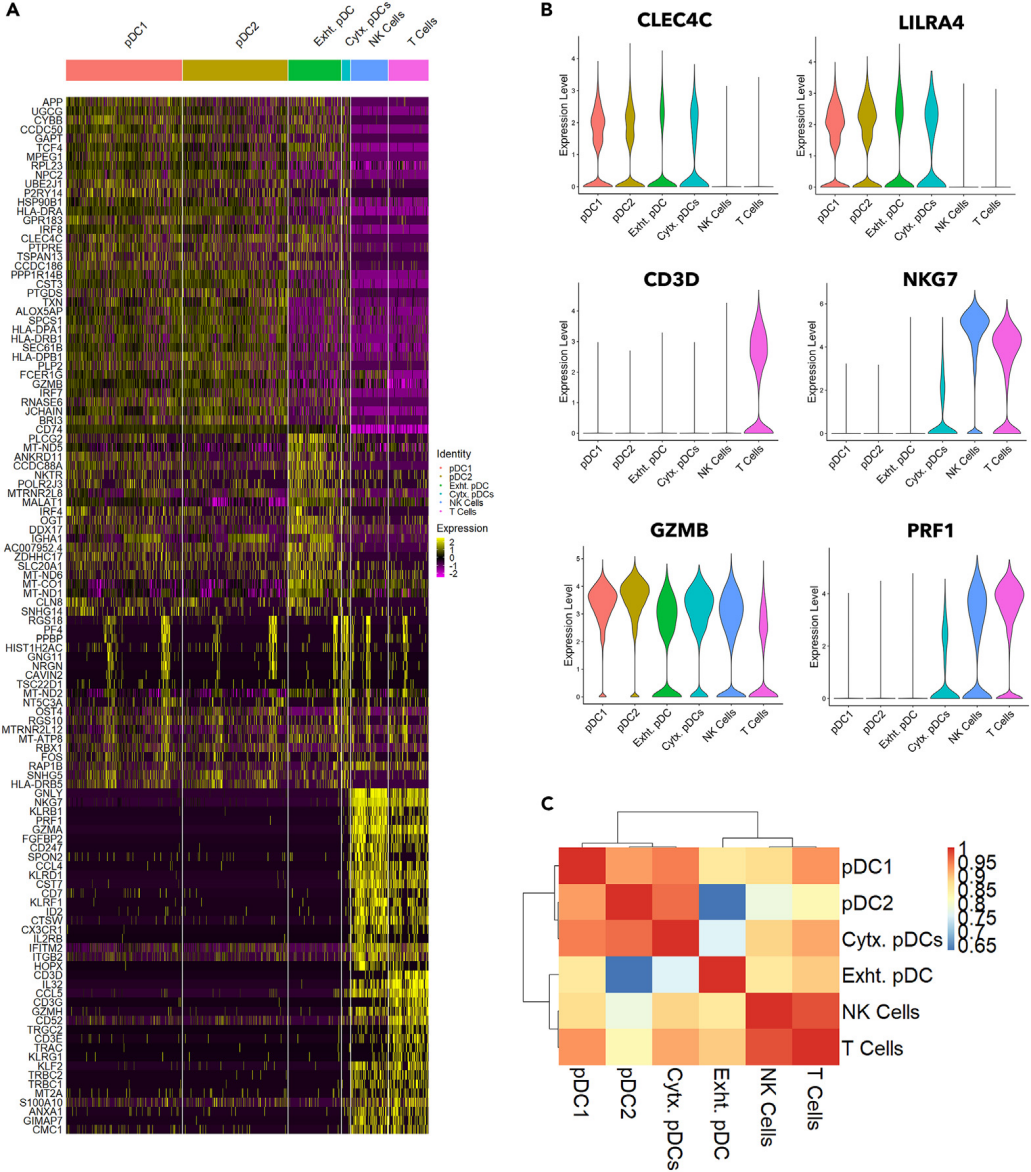
Cytotoxic-like pDCs are discrete from NK and T cells pDC1, pDC2, cytotoxic-like pDC (Cytx. pDC), exhausted pDC (Exht. pDC), NK cells and T cells clusters were selected from the main dataset, integrated and analyzed. (A) Heatmap representation of top20 genes from each subset. (B) Violin plot representation of expression level of *CLEC4C, LILRA4, CD3D, NKG7, GZMB* and *PRF1* in pDC clusters, NK cells and T cells. (C) Heatmap representation of cell types transcriptional correlation analysis of cytotoxic-like pDCs to other pDC clusters, NK cells and T cells.

Since the described cytotoxic-like pDC subset shares expression of several cytotoxic genes with NK and T cells (e.g., NKG7, granzyme B and perforin^29^^,^^30^), we sought to understand how cytotoxic-like pDCs differ from cytotoxic NK and T cells. To this end, we annotated and integrated datasets from pDC1, pDC2, cytotoxic-like pDC, exhausted pDC cluster (Figure 2A), and NK cells and T cells (Figure S2A), and then analyzed the top 20 genes in each subset. While cytotoxic-like pDCs expressed all known classical pDCs genes and T and NK cells expressed their known gene signatures (Figure 3A), cytotoxic-like pDCs had lower expression of cytotoxic genes such as *NKG7* and *PRF1* relatively to NK cells and T cells (Figure 3B). Additionally, the cluster correlation analysis showed a lower correlation between cytotoxic-like pDCs with NK cells and T cells (Figure 3C). Collectively, our data confirmed that despite the shared expression of several cytotoxic genes, cytotoxic-like pDCs are clearly of pDC origin and they are distinct from NK cells and T cells.

### Cytotoxic-like pDCs exhibit similar cell to cell communication as other pDC subsets

**Figure 4:**
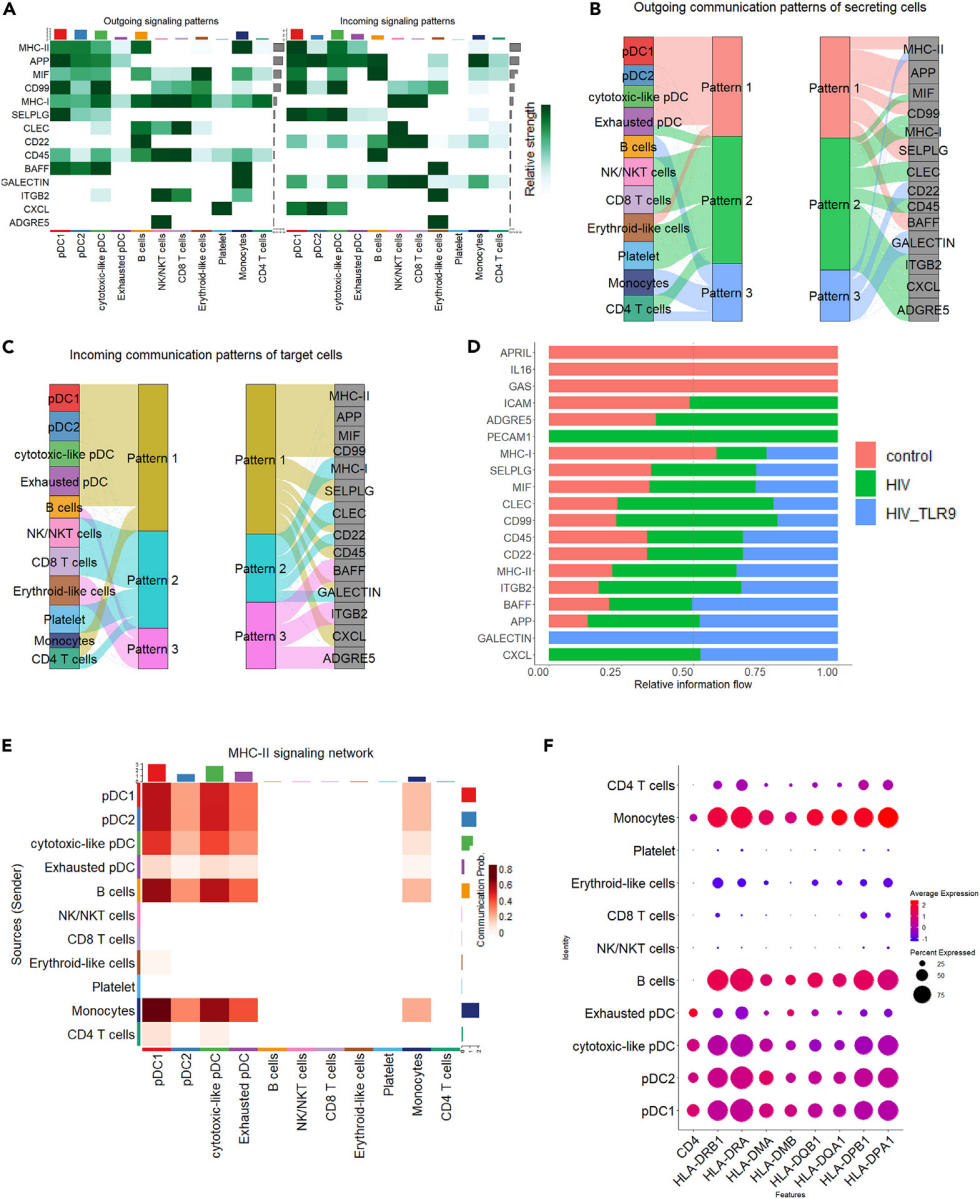
Cytotoxic-like pDCs exhibit similar cell to cell communication as other pDC clusters (A) Heatmap illustration of the inferred intercellular communication networks of most significant signaling between different cell types either by their incoming or outgoing signals. (B and C) (B) The inferred outgoing and (C) incoming communication pattern of secreting and target cells which shows the correspondence between the inferred latent pattern, cell groups as well as signaling pathways. (D) Information flow of significant signaling pathway within the inferred network between healthy individuals, PWH at baseline and during TLR9 agonist treatment. (E) Heatmap illustration of MHC-II signaling pathway among different cell types. (F) Dot plot representation of average and percentage of gene expression of HLA-D isotype and CD4.

To determine cell-to-cell communication between the cytotoxic-like pDC cluster and the other pDC clusters, we employed CellChat analysis which enables analyses of cellular communication networks.^31^ For this analysis, we integrated datasets from healthy individuals and PWH both at baseline and during TLR9 agonist treatment. Following manual annotation of cell clusters using their differentially expressed genes, we identified main 11 clusters: pDC1, pDC2, cytotoxic-like pDCs, exhausted pDCs, B cells, NK/NKT cells, CD8^+^ T cells, CD4^+^ T cells, platelets, erythroid-like cells and monocytes (Figures S3A and S3B). A global communication analysis based on ligand-receptor/co-receptor pairs revealed that communication through signaling pathways such as *MHC-II, MHC-I, APP, MIF, CD99, SELPLG, CLEC, BAFF, CD22, GALECTIC, ITGB2, ADGRES, and CXCL* were the most significant signaling pathways (Figures 4A and S3C). Next, we used a pattern recognition method analysis to identify the global communication patterns and key signals for different cell types. The pattern recognition method is based on non-negative matrix factorization and the outcome of this analysis is a set of communication patterns that connect cell types with signaling pathways. Thus, this method allowed us to connect cell types with signaling pathways either in the context of incoming or outgoing signaling. We found that *MHC-II, APP, MIF*, *BAFF*, and *CD99* communicating signaling were predominant among pDC subsets. We further observed that the cytotoxic pDC cluster shared similar cellular incoming and outgoing signaling with other pDC clusters (Figures 4B, 4C, and S3D). To understand how HIV infection and TLR9 agonist treatment may impact cell communication signaling, we compared the information flow defined as the sum of communication probabilities for these signaling pathways, between healthy individuals, PWH at baseline and during TLR9a treatment. We found that cell-to-cell communication by *MHC-II, MHC-I, APP, MIF, SELPLG, and CD99* was similar for the three conditions. However, *APRIL, IL16,* and *GAS* signaling pathways were found only in healthy individuals, PECAM1 in PWH at baseline, and *GALECTIN* in HIV during TLR9 agonist treatment (Figure 4D). Furthermore, we analyzed ligand-receptor interaction of some of these signaling pathways. The MHC-II signaling pathway has mostly been described between antigen-presenting cells (including pDCs) and CD4^+^ T cells but notably, pDCs express both MHC-II ligand and CD4 receptor.^32^^,^^33^ Our data showed several interactions between HLA-D isotypes with CD4 receptor within pDCs and further suggested that pDCs can both send and receive the MHC-II ligand to the CD4 receptor (Figure S3D). When we further characterized MHC-II signaling pathway, we found a strong MHC-II - CD4 interaction within pDCs (Figure 4E). We also observed that all pDC clusters expressed most HLA-D isotypes and CD4 receptor. Of note, pDCs expressed considerably higher levels of CD4 than CD4^+^ T cells (Figure 4F). In addition to MHC-II signaling, CD99 - CD99 communication signaling was stronger between pDC1 and cytotoxic-like pDC cluster than between the other pDC clusters (Figure S3E) and only pDC1 and cytotoxic-like pDC clusters were shown to receive the MIF ligand to their CD74 or CXCR4 receptors (Figure S3F). Collectively, we observed previously unknown ligand-receptor interactions within pDCs, such as MHC-II – CD4, MIF – CD74/CXCR4 and CD99 – CD99. Our data also demonstrate that despite their cytotoxic transcriptomic profile, cytotoxic-like pDCs exhibit similar cellular incoming and outgoing cell-to-cell communication as other pDCs, further supporting that cytotoxic-like pDCs are truly pDCs.

## Discussion

Our study provides comprehensive transcriptomic profiling of gene expression of over 112,000 pDCs not only from healthy individuals but also from PWH both before and after 4 weeks of TLR9 agonist treatment. Our data revealed that despite many years of successful ART treatment and suppressed plasma HIV RNA, pDCs from PWH exhibit a pronounced upregulation of immune regulatory genes compared to healthy individuals. Furthermore, we identified two homogeneous pDCs subsets (pDC1 and pDC2), an exhausted pDC subset and a cytotoxic-like pDC subset. This cytotoxic-like pDC subset is characterized by relatively higher expression of genes associated with cellular antiviral and cytotoxic activity. Nevertheless, a comparative gene expression analysis showed that these cytotoxic-like pDCs express classic pDC genes and are clearly distinguishable from NK and T cells despite their common expression of several cytotoxic genes. Finally, our cell communication analysis showed that cytotoxic-like pDCs shared similar incoming and outgoing communication signaling like other pDC subsets confirming that they are indeed pDCs.

Prior studies on the role of pDCs in HIV infection focused mainly on IFN-I production and antiviral ISGs.^34^^,^^35^^,^^36^^,^^37^ However, none of these studies provided in-depth profiling of single pDCs. By comparing PWH on ART to healthy individuals, our study findings were not limited to differences in IFN-I pathway regulation, but also identified differential regulation of other genes involved in cytokine regulation and production, complement activation, antigen presentation, NK and T cell activation Furthermore, we also observed that TLR9 agonist treatment during HIV infection induced upregulation of select genes involved in pDCs cytokine and chemokine production as well as activation and function of adaptive immune cells, consistent with earlier reports in HIV^26^^,^^38^ and cancer studies.^39^^,^^40^

Our transcriptomic profiling of pDC subsets contrast the traditional flow cytometry-based classification of pDCs subsets by their surface expression of CD2. A previous single cell study of dendritic cells reported that CD2^hi^ pDCs appeared to correspond to AS dendritic cells.^27^ Our classification of pDCs in to pDC1, pDC2, exhausted pDCs and cytotoxic-like pDCs was based on the phenotypic expression of genes commonly associated with pDCs. Although pDC1 and pDC2 shared similar expression signatures and localized in neighboring homogeneous clusters, the expression of *IRF7*, *MZB1*, and *LILRA4* aided in distinguishing the two subsets. The higher relative expression of *IRF7, MZB1* and *LILRA4* may suggest that pDC2s produce more IFN-I than pDC1s. In contrast, exhausted pDCs had high expression of genes associated with mitochondrial stress, cellular activation, and exhaustion, strongly indicating that this pDC subset consists of terminally exhausted and dying pDCs.

Cytotoxic properties are essential for effector cell-mediated killing of infected and tumor cells - a cellular characteristic primarily ascribed to NK cells, CD8^+^ T cells and a subset of CD4^+^ T cells. In the present study, we identified a new subset of cytotoxic-like pDCs which expressed multiple molecules associated with cytotoxicity in NK cells and T cells while also expressing classical pDC phenotypic markers. Our findings extend earlier reports suggesting that dendritic cells (including pDCs) may to some extent have cytotoxic properties. For instance, Plitas et al. and Chastain et al. reported a new innate immune subset that expresses phenotypic markers and functional activities of both NK cells and dendritic cells called NKDCs. These NKDCs were shown to be cytotoxic, antigen presenting and able to produce IFN-γ.^41^^,^^42^ Furthermore, other studies have reported that surface protein expression of the effector molecule TRAIL on pDCs endow them with killing properties. Among untreated people with HIV that are viremic, a subset of activated pDCs is transformed into TRAIL expressing IFN-producing killer pDCs and these TRAIL^+^ pDCs can kill CD4^+^ T cells.^23^^,^^24^^,^^25^ Additionally, activated pDCs can lyse certain melanoma cell lines in a TRAIL-dependent manner.^25^ However, the cytotoxic-like pDC subset identified in our study was not confined by high TRAIL (TNFSF10) expression but also co-expressed multiple antiviral and cytotoxic genes including *MX1, ISG15, OAS3, NKG7, GZM k, GNLY*, perforin, and *KLRB1*.

To understand how cytotoxic-like pDCs communicate with other circulating immune cells, we employed a newly developed Cell-Cell communication toolkit called CellChat. Our results showed that cytotoxic-like pDCs have the same incoming and outgoing communicating signaling pathways with that of other pDC subsets confirming that they are indeed pDCs. However, we observed several potential novel ligand-receptor interactions within pDC subsets that include MHC-II interaction to CD4 receptor, CD99 interaction to CD99, and APP interaction to CD74. Of note, pDCs express both the MHC-II and CD4 receptor, yet little is known about the function of the CD4 receptor on pDCs or the interaction between MHC-II and CD4 receptor on pDCs. Whether the interactions of MHC-II and CD4 receptor affects pDCs phenotype and function is also not known but will be important to address in future studies.

In summary, our pDC transcriptome profiling elucidated differences in gene expression patterns between healthy individuals and people with HIV infection. We also identified a novel cytotoxic-like pDC subset and revealed novel ligand receptor interactions within pDCs. While the mechanisms of induction, regulation and function of these cytotoxic-like pDCs remain unknown, our study will serve as a reference for future studies.

### Limitations of the study

The size of the study population was small which, as noted above, limited our ability to identify clinical characteristics that may be associated with the individual composition of the pDC compartment. Also, due to the absence of women among PWH, we could not analyze gender specific differences. Finally, while further investigations on the mechanism of induction and killing function of these cytotoxic-like pDCs are essential, functional studies of pDCs are very challenging. First, pDCs only make up 0.1 to 0.5% of all PBMCs and large blood volumes are required to obtain enough cells for the experiments. Second, functional experiments need to be done on freshly isolated PBMCs because frozen cryopreserved pDCs respond very poorly when thawed. Lastly, pDCs limited time in circulating blood poses another challenge with regards to tracking and determining the mechanisms that regulate the homeostasis of cytotoxic-like pDCs.

## STAR★Methods

### Key resources table

**Table:** 

REAGENT or RESOURCE	SOURCE	IDENTIFIER
**Antibodies**		

Chromium Next GEM Single Cell 3’ Kit v3.1	10x Genomics	PN-1000268
3’ Feature Barcode Kit	10x Genomics	PN-1000262
Chromium Next GEM Chip G Single Cell Kit	10x Genomics	PN-1000120
Dual Index Kit TT Set A	10x Genomics	PN-1000215
Dual Index Kit NN Set A,	10x Genomics	PN-1000243
Dual Index Kit NT Set A,	10x Genomics	PN-1000242
EasySep Dead Cell Removal (AnnexinV) Kit	STEMCELL	Cat #:17899
Human plasmacytoid dendritic cells isolation kit II	Miltenyi Biotec	Cat #:130-092-207
Fixable Near IR	Thermo Fisher	*Cat #: L10119*
Anti-Lin Cocktail	Biolegend	Cat #: 348801
Anti-CD303	Thermo Fisher	Cat #: 25-9818-42
Anti-CD123	Thermo Fisher	Cat #: 20-0116

**Deposited data**		

Raw and processed sc-RNA-seq data	This paper	GEO accession GSE228078
Codes for downstream analysis	This paper	https://github.com/laminbcham/sc-RNA-of-human-pDC-in-HIV-infection/blob/main/figure%20analysis%20codes

**Software and algorithms**		

R studio version 4.0.3		https://www.r-project.org/
Cell ranger count version 3.0.2	10X Genomics	https://support.10xgenomics.com/singlecell-geneexpression/software/downloads/3.0/
Seurat version 4.0.3	Seurat	https://cran.rproject.org/web/packages/Seurat/index.html
FlowJo software	FlowJo LLC, Ashland, OR, USA	FlowJo LLC

### Resource availability

#### Lead contact

Individual participant data cannot be made available due to EU Data Protection Regulations (GDPR). A limited and completely anonymized version of the dataset can be obtained upon request. Further information and requests for resources and reagents should be directed to and will be fulfilled by the Lead Contact: olesoega@rm.dk.

#### Materials availability

This study did not generate new unique reagents.

### Experimental model and study participant details

The biological samples used in this study are from eight participants (7 men, 1 woman), median age is 37,5 years (age range is 24 – 70 years) and all participants are white/Caucasian as shown in detailed in Table 1. ThePWH were enrolled in the TEACH clinical trial.^26^ The study was approved by the National Health Ethics Committee, Denmark (case number 1-10-72-10-15), the Danish Medicines Agency (case number 2015014125), and the Danish Data Protection Agency. The trial was monitored in accordance with the principles for good clinical practice. Each patient provided written informed consent prior to any study procedures. Study participants received 4 weeks of 60 mg (concentration 15 mg/mL) of MGN1703 (MOLOGEN AG, Berlin, Germany), administered subcutaneously by the study investigator as two 2-mL bilateral injections twice weekly. ART was maintained during the entire study period and blood samples were collected at baseline (before first dose of TLR9 agonist treatment) and during the 4th week of treatment. PBMC were immediately isolated and stored in liquid nitrogen.^26^

### Method details

#### Dead cell removal

PBMCs were thawed and cells were suspended in to 1-2 ML of buffer and used 30 μm pre-separation filter. For dead cell removal, EasySep Dead Cell Removal kit (Catalog # 17899 STEMCELL Technologies) was used. Cell pellets were resuspended in 400ul of 1× Binding Buffer,100 μL of Dead Cell Removal Microbeads were added, mixed and incubated for 15 minutes at room temperature (20−25°C). Cells were resuspended and proceeded to magnetic separation.

#### pDC negative isolation

The human plasmacytoid dendritic cells isolation kit II (Cat. # 130-092-207, Miltenyi Biotec) was used. Sorted live cells were resuspended in 400 μL of buffer, 100 μL of the Non-PDC Biotin-Antibody Cocktail II was added and the cell suspension was incubated for 10 minutes in the refrigerator (2−8°C). Cells were washed, 400 μL of buffer and 100 μL of the Non-PDC Microbead Cocktail II were added and the solution was incubated for an additional 15 minutes in the refrigerator (2−8°C). Cells were resuspended in 500 μL of buffer and proceeded to magnetic separation using the LD Column in the magnetic field of a suitable MACS Separator. The sorted cells were counted, flow cytometry with CD303 and CD123 was performed, and then we proceeded to single cell RNA library preparation.

#### Single cell RNA library prep and sequencing

Briefly after pDCs were isolated, cells and reagents were prepared and loaded into the chip and ran into the Chromium Controller for Gel Bead-In Emulsion (GEM) generation and barcoding. The input number of cells was estimated at 15-20,000 cells per sample. The Chromium Next GEM Single Cell 3’ Gel beads v3.1 kit (10X Genomics, Pleasanton, CA, USA) was used to create GEMs following manufacturer’s instruction. All GEMs generated were used for cDNA synthesis and library preparation using the Chromium Single Cell 3’ Library Kit v3.1 (10X Genomics) following the manufacturer’s instruction. scRNA-seq libraries were then prepared using GemCode Single Cell 3′ Gel bead and library kit (10X Genomics) following the manufacturer’s instruction. cDNA concentration of each sample was measured using a Tapestation 2200 system (Agilent). Single-cell barcoded cDNA libraries were sequenced on an Illumina Illumina Novaseq6000 system (100-cycle cartridge) with a sequencing depth of at least 50,000 reads per cell.

#### Single cell RNA-Seq data pre-processing

For the preprocessing, individual raw scRNAseq fastq files form each sample were aligned against the human reference genome (GRCh38) through the cell ranger count pipeline (Cell Ranger version 3.1.0, 10x Genomics Technology), which generated the cell-genes count matrices. For data clean up, quality control and analysis, we used R studio and the Seurat package (version 4.0). We performed a clean-up and quality control based on cellular expression of mitochondrial genes (cells with a percentage of mitochondrial genes >25% were discarded). To be considered for further analysis, genes had to be expressed in more than ten cells, cellular barcodes had to be associated to at least 200 genes. We next identified doublets using the DoubletFinder algorithm and removed these cellular barcodes from the analysis. Each singlet file for each sample was saved and later integrated for further analysis.

#### Data integration and selection of pDCs

After quality control, removing cell doublets and filtering out low quality cells, we normalized sc-RNA-seq dataset from each sample separately to generate individual dataset. To perform comparison between samples from healthy controls and PWH before and during TLR9 agonist treatment, using the ‘‘FindIntegrationAnchors’’ Seurat function, we first integrated singlet data of samples from HIV baseline and healthy controls. We then integrated data of samples from PWH at baseline and during TLR9 agonist treatment. Next, we normalized each integrated dataset and performed PCA and cluster analysis with a cluster resolution of 0,3. To assign cellular identity, we applied graph-based clustering and a non-linear dimension reduction using uniform manifold approximation and projection (UMAP) for cell cluster visualization. Using the differentially expressed genes for known lineage markers, we annotated cell types based on these markers: pDC cluster (*CLEC4C, LILRA4, IL3RA*), monocytes (*LZY*), B cells (*MS4A1*), NK cells, (*GNLY, NKG7*), T cells (*CD3G, CD3D*), platelet (*PPBP*), and erythroid like cells (*IGHA1, IGKC, IGLC2*). We annotated clusters (as shown in Figures S1B, S1C, S2A, and S2B) and the pDC cluster from each integrated dataset was selected for further downstream analysis (Figures 1 and 2). The re-clustering of the pDC yielded three clusters and we annotated these clusters, once again based on relative expression level of differentially expressed genes. The comparison between clusters was performed by calculating the Pearson correlation between the averaging normalized gene expression of each cluster. Using the “AverageExpression” function, we calculated and visualized relative gene expressions on a heatmap plot. Next, we set our “Idents” as ‘condition’ (healthy control, HIV-1 or HIV+TLR9a) and ran the differential expression analysis between these groups using the ‘FindAllMarkers’ Seurat function. The differentially expressed genes among groups were visualized on a volcanos plot and Gene Ontology (GO) analysis was run using the clusterProfiler package.

#### Cellchat analysis

sc-RNA-seq analysis toolkits such as CellChat have a constructed database of interactions among ligands, receptors and co-receptors and can predict major signaling input and output thereby helping in the discovery of novel intercellular communications. Furthermore, CellChat enables to quantitatively infer and analyze cellular communication network.^31^ Cell–cell communication network was performed by integrating all three groups of samples and visualized using the “netVisual_aggregate” function. The centrality score was computed and visualized using the “netAnalysis_signalingRole_network” function while relative contribution of each ligand-receptor pair was visualized using the “netAnalysis_contribution” function as described in Wang et al.^43^ The global communication patterns analysis was computed using ‘’identifyCommunicationPatterns’’ and visualized using ‘’netAnalysis_river’’ function. Application of this analysis uncovered three patterns for outgoing signaling and 3 patterns for incoming signaling either in the context of outgoing signaling (as senders) or incoming signaling (as receivers). Heatmap plots were computed and visualized using ‘’netVisual_heatmap’’ function.

### Quantification and statistical analysis

Differential expression genes (DEGs) were analyzed using three tests, Wilcoxon-ranked sum test, t-test and t-test overestimated variance. DEGs were computed using the ‘FindMarker’ function of Seurat and the probability values were estimated with respect to all other clusters within each dataset.

### Additional resources

Samples used in this study are from participants enrolled in the TEACH clinical trial. The study was approved by the National Health Ethics Committee, Denmark (case number 1-10-72-10-15), the Danish Medicines Agency (case number 2015014125), and the Danish Data Protection Agency.

## Data Availability

• Single cell RNA-seq raw and processed data have been deposited at Gene Expression Omnibus (GEO accession GSE228078) https://www.ncbi.nlm.nih.gov/geo/query/acc.cgi?acc=GSE228078. • Original codes used to generate data of this paper are publicly available at GitHub: https://github.com/laminbcham/sc-RNA-of-human-pDC-in-HIV-infection/blob/main/figure%20analysis%20codes. • Any additional information required to reanalyze the data reported in this paper is available from the lead contact upon request.
