# Supplementary material for: Single cell analysis reveals a subset of cytotoxic-like plasmacytoid dendritic cells in people with HIV-1
Source: iScience. 2023 Aug 12;26(9):107628. doi: 10.1016/j.isci.2023.107628 (PMC10470411; doi:10.1016/j.isci.2023.107628)
Supplement: Document S1. Figures S1–S3 [file mmc1.pdf]

iScience, Volume *26*

Supplemental information

Single cell analysis reveals a subset of cytotoxic-like plasmacytoid dendritic cells in people with HIV-1

Lamin B. Cham, Jesper D. Gunst, Mariane H. Schleimann, Giacomo S. Frattari, Miriam Rosas-Umbert, Line K. Vibholm, Renée M. van der Sluis, Martin R. Jakobsen, Rikke Olesen, Lin Lin, Martin Tolstrup, and Ole S. Søgaard

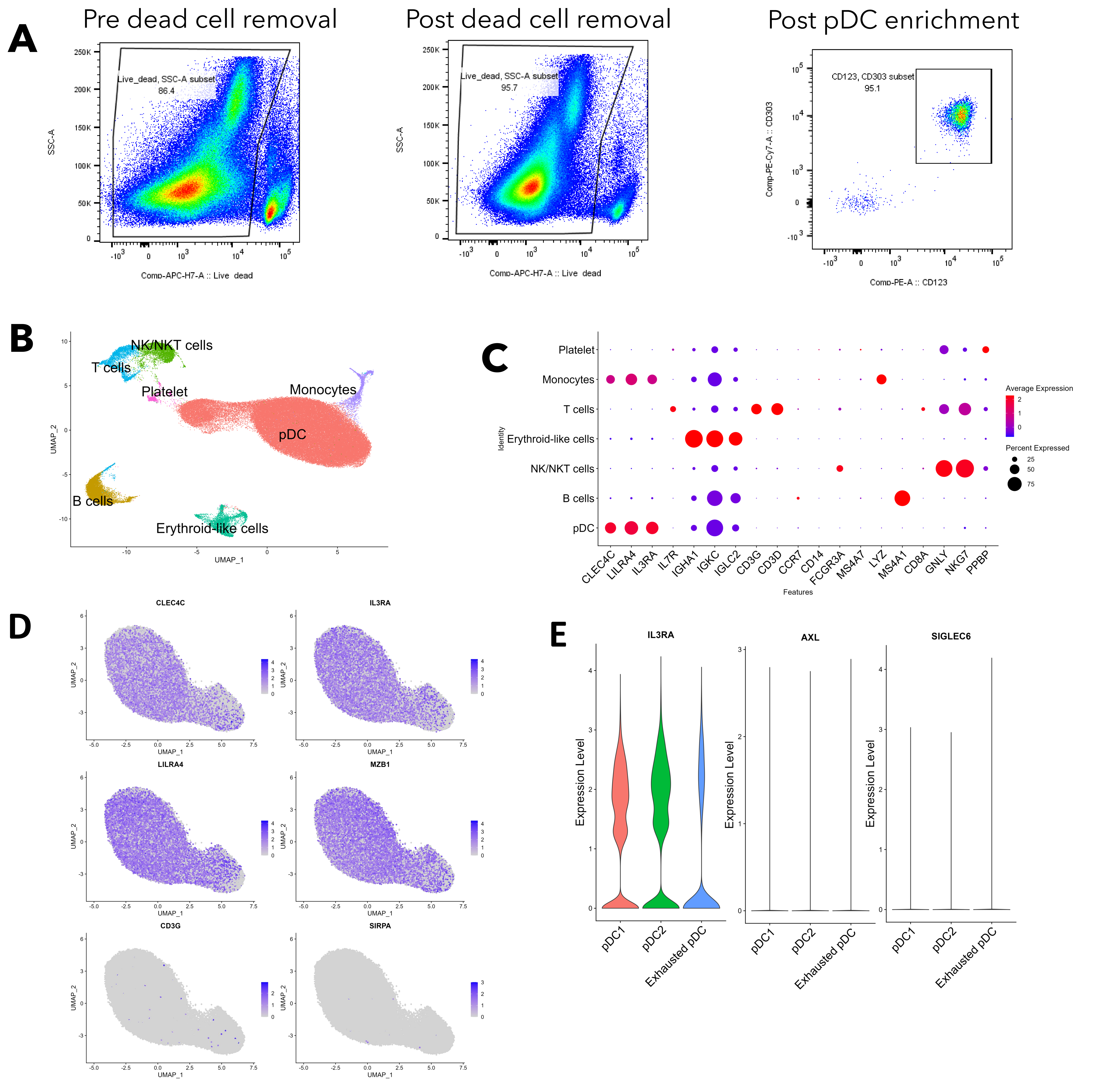

Figure S1: Gating strategies for single cell sorting and cluster annotation, related to figure 1

(A) Illustration of dead cell removal and pDC isolation outcome by flow cytometry. Individually generated sc-RNA sequence data were preprocessed and sc-RNA singlet data from four healthy and four HIV-1 individuals at baseline were integrated into a single dataset for downstream analysis. (B) UMAP display of main pDC cluster and contaminants of other immune cells. (C) Dot plot representation of average and percentage expression of gene signatures of each cell type. The main pDC cluster was selected for further in-depth analysis. (D) Feature plot showing expression level of *CLEC4C, IL3RA, LILRA4, MZB1, CD3G* and *SIRPa* on pDCs and (E) violin plot representation of *IL3RA, AXL* and *SIGLEC6* among pDC clusters.

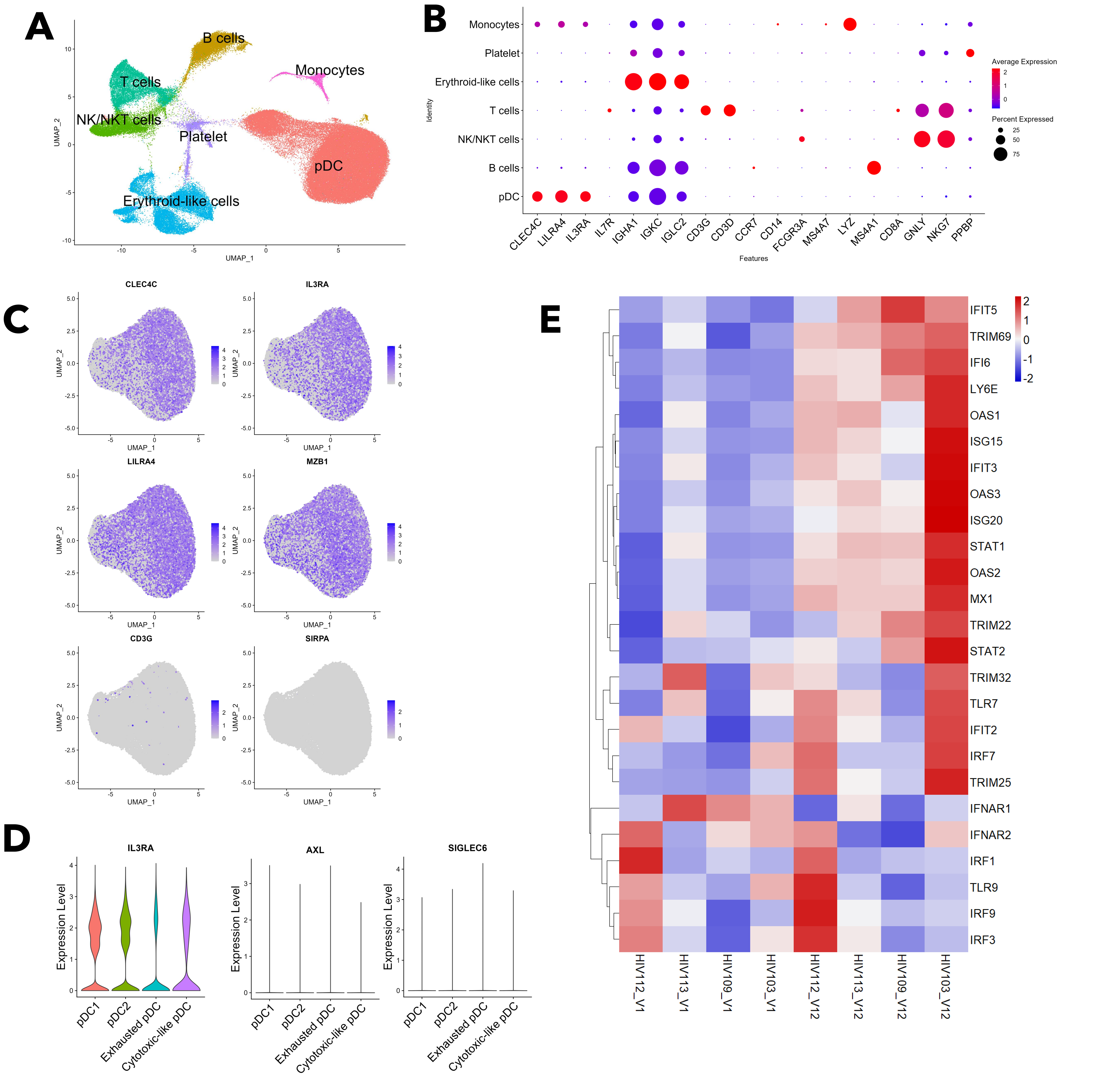

Figure S2: Cluster annotation and gene expression analysis , related to figure 2

Individually generated sc-RNA sequence data were preprocessed and four sc-RNA singlet data from HIV baseline and during TLR9 agonist treatment samples were integrated into a single dataset for comparative analysis. (A) UMAP display of main pDC cluster and contaminants of other immune cells. (B) Dot plot representation of average and percentage expression of gene signature of each cell type. The main pDC cluster was selected for further analysis. (C) Feature plot showing expression level of *CLEC4C, IL3RA, LILRA4, MZB1, CD3G* and *SIRPa* on pDCs and (D) violin plot representation of *IL3RA, AXL* and *SIGLEC6* among pDC clusters. (E) Heatmap representation of relative gene expression of IFN-I and other immune-modulatory genes of samples from PWH baseline and during TLR9 agonist treatment.

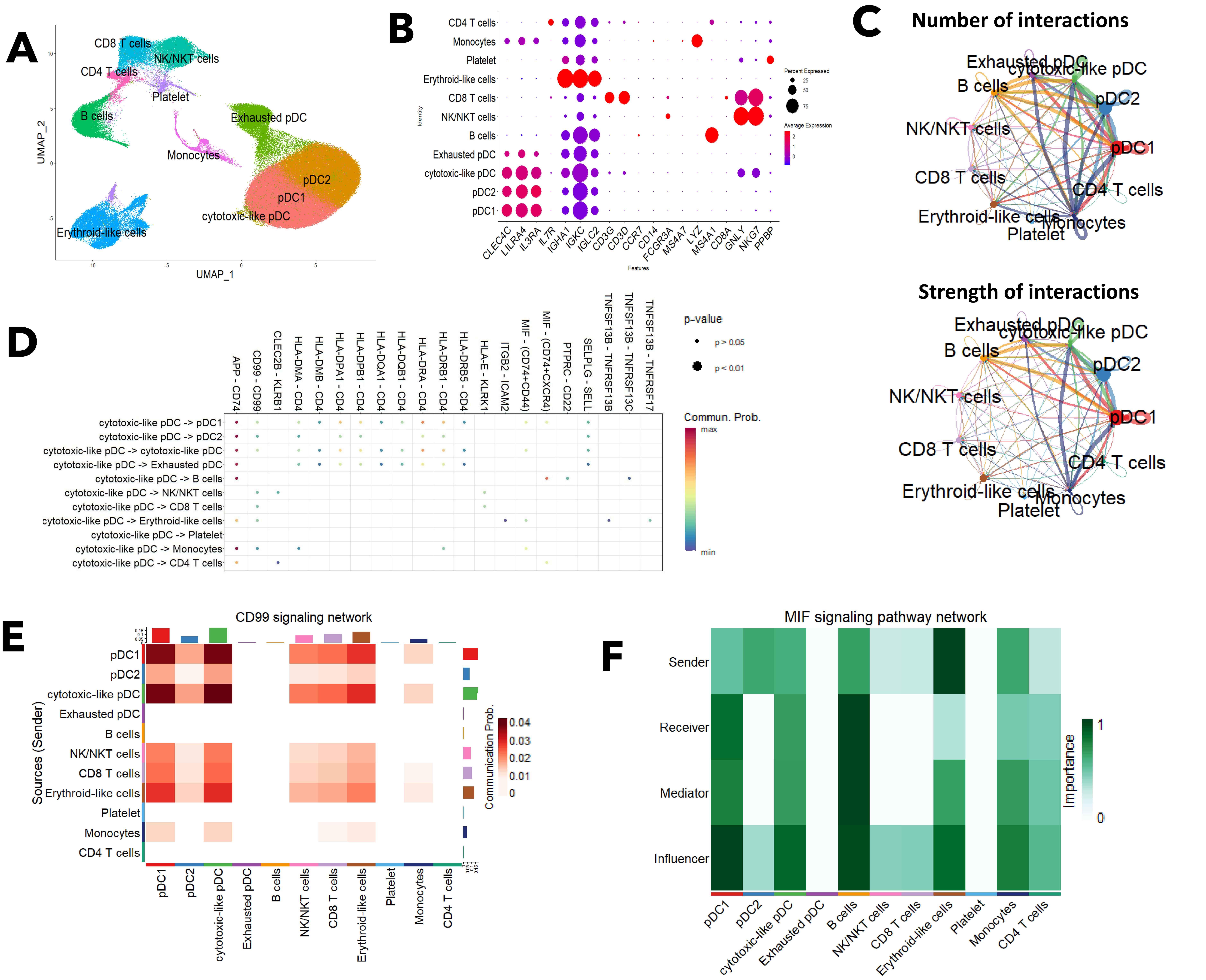

Figure S3: Cluster annotation and cell communication analysis , related to figure 4
(A) UMAP illustration of immune cell type of integrated dataset from healthy individuals, HIV baseline and during TLR9 agonist treatment and (B) Dot plot representation of average and percentage expression of gene signatures of each cell type. (C) Comparison of cell-to-cell communication by number and strength of interactions between cell types. (D) Ligand-receptor interaction of each pDC cluster against each other and other immune cell types. (E) Heatmap representation of cellular communication of CD99 signaling pathways and (F) MIF signaling pathways network.
